# Indirect comparison of the diagnostic performance of ^18^F-FDG PET/CT and MRI in differentiating benign and malignant ovarian or adnexal tumors: a systematic review and meta-analysis

**DOI:** 10.1186/s12885-021-08815-3

**Published:** 2021-10-06

**Authors:** Xianwen Hu, Dandan Li, Zhigang Liang, Yan Liao, Ling Yang, Rui Wang, Pan Wang, Jiong Cai

**Affiliations:** 1 Department of Nuclear Medicine, Affiliated Hospital of Zunyi Medical University, No.149 Dalian Road, Huichuan District, Zunyi, Guizhou Province PR China 563003; 2 Department of Radiology, Weng ‘an Qingzhu Hospital, Weng ‘an, Guizhou PR China 540400; 3 Department of Obstetrics, Zunyi Hospital of Traditional Chinese Medicine, Zunyi, Guizhou PR China 563003

**Keywords:** Ovarian tumor, Positron emission tomography/computed tomography, Magnetic resonance imaging, Meta-analysis

## Abstract

**Objective:**

To compare the value of fluorodeoxyglucose positron emission tomography (FDG-PET)/computed tomography (CT) and magnetic resonance imaging (MRI) in differentiating benign and malignant ovarian or adnexal tumors.

**Materials and methods:**

English articles reporting on the diagnostic performance of MRI or ^18^F-FDG PET/CT in identifying benign and malignant ovarian or adnexal tumors published in PubMed and Embase between January 2000 and January 2021 were included in the meta-analysis. Two authors independently extracted the data. If the data presented in the study report could be used to construct a 2 × 2 contingency table comparing ^18^F-FDG PET/CT and MRI, the studies were selected for the analysis. The Quality Assessment of Diagnostic Accuracy Studies-2 (QUADAS-2) was used to evaluate the quality of the included studies. Forest plots were generated according to the sensitivity and specificity of ^18^F-FDG PET/CT and MRI.

**Results:**

A total of 27 articles, including 11^18^F-FDG PET/CT studies and 17 MRI studies on the differentiation of benign and malignant ovarian or adnexal tumors, were included in this meta-analysis. The pooled sensitivity and specificity for ^18^F-FDG PET/CT in differentiating benign and malignant ovarian or adnexal tumors were 0.94 (95% CI, 0.87–0.97) and 0.86 (95% CI, 0.79–0.91), respectively, and the pooled sensitivity and specificity for MRI were 0.92 (95% CI: 0.89–0.95) and 0.85 (95% CI: 0.79–0.89), respectively.

**Conclusion:**

While MRI and ^18^F-FDG PET/CT both showed to have high and similar diagnostic performance in the differential diagnosis of benign and malignant ovarian or adnexal tumors, MRI, a promising non-radiation imaging technology, may be a more suitable choice for patients with ovarian or accessory tumors. Nonetheless, prospective studies directly comparing MRI and 18F-FDG PET/CT diagnostic performance in the differentiation of benign and malignant ovarian or adnexal tumors are needed.

**Supplementary Information:**

The online version contains supplementary material available at 10.1186/s12885-021-08815-3.

## Introduction

Ovarian cancer is the disease with the highest mortality rate among malignant tumors affecting the female reproductive tract. According to available statistics, more than 180 thousand women die of ovarian cancer every year worldwide [1]. Ovarian cancer is highly heterogeneous and can be classified into epithelial tumors, germ cell tumors, sex cord-stromal tumors, and other tumors [2]. Among these, epithelial ovarian cancer accounts for 90% of all cases [3]. The main risk factors for ovarian cancer are family genetic history, fertility factors, menstrual history, breastfeeding, height and body mass index, contraception, exercise, lifestyle, diet, gynecological diseases, psychological factors, and hormone replacement therapy [4–8]. Some studies suggested that smoking, a high-fat diet, ionizing radiation, talcum powder, and ABO blood type are also risk factors for ovarian cancer [9–11]. Moreover, Ness et al. have suggested that height (≥1.7 m) and body mass index ≥30 kg/m2 are also high-risk factors, while tubal ligation and short-term use of intrauterine devices can reduce the risk of ovarian cancer [10]. Moreover, Braem et al. [7] found that ovarian cancer was negatively correlated with increased parity, prolonged use of oral contraceptives, hysterectomy, younger age at natural menopause, exercise time, and annual shortening of menstrual cycles.

Ovarian cancer is an aggressive type of tumor. As no specific clinical symptoms are found in the early stage, diagnosis may be challenging. Mathieu et al. found that only 20–25% of ovarian cancer patients can be correctly diagnosed in the early stage [12]. Moreover, another study reported that about 60% of ovarian cancer patients are at an advanced stage at the time of diagnosis; these patients often have a poor prognosis and a low 5-year survival rate (below 30%) [13]. Therefore, developing a highly sensitive and specific diagnostic method may be crucial for early diagnosis, clinical staging, guiding treatment, and improving ovarian cancer prognosis.

Serological tumor markers and imaging methods, such as ultrasound, CT, MRI, and PET/CT, are the most common diagnostic approaches for ovarian cancer. Serum carbohydrate antigen (CA)125 is the most widely studied biomarker for ovarian cancer [14]. CA125 is elevated in 75% of patients with early-stage ovarian cancer, but its specificity is only 70.61% [5, 6]. CA125 is also positively expressed in other malignant tumors, including lung cancer, colorectal cancer, endometrial cancer, breast cancer, and lymphoma. Moreover, the expression level of CA125 is also increased in common pelvic benign diseases, such as adnexal cyst, endometriosis, uterine fibroids, and pelvic inflammatory disease [15]. In addition to CA125, recent studies have shown that HE4, a serum marker for ovarian cancer, has a specificity of more than 95% and a sensitivity of 70% [16, 17]. Moreover, carcinoembryonic antigen (CEA), gonadal hormone, CA72–4, CA15–3, and alkaline phosphatase have also been used as serum markers for ovarian cancer, but their sensitivity for detecting ovarian cancer is lower than 75% [17].

Ultrasound is a commonly used imaging method for gynecological diseases due to its simplicity and non-radiation exposure. However, the small size of early ovarian cancer may be limited to the ovary and does not cause ovarian morphological changes, which can lead to false-negative results. Moreover, differentiating ovarian cancer from ovarian cystadenoma, immature teratoma, and other diseases using ultrasound may be challenging [18]. On the other hand, CT and MRI can provide the anatomical information of the ovarian and its surrounding tissues, which are of great clinical significance for determining the scope of invasion of ovarian cancer and the formulation of surgery plans. MRI is a biological magnetic spin imaging technology that uses the hydrogen atoms in the human body all over the body to be excited by radio frequency pulses in an externally strong magnetic field to produce nuclear magnetic resonance. After spatial coding technology, the detector detects and receives the nuclear magnetic resonance signal emitted in the form of electromagnetic, input it into the computer, after data processing and conversion, and finally the shape of the human body tissues is formed into an image for diagnosis [19]. MRI is superior to CT in soft tissue resolution but may not be enough when detecting tumors smaller than 5 mm [20]. PET/CT imaging integrates CT and PET to achieve integration, organically integrating anatomical imaging and functional imaging, which can clearly and intuitively reflect the changes in tumor cell metabolism, so as to accurately and early diagnose tumors [21]. Most malignant tumor cells have strong metabolism and corresponding increase in energy consumption. Glucose is one of the main energy sources of tissue cells, and ^18^F-FDG can reflect the glucose utilization status of normal tissues of the body. Therefore, compared to MRI and CT, ^18^F-FDG PET/CT imaging can show both structural and functional data of the tumor and is often used to examine tumor cells at the molecular stage, which leads to positive manifestations of high metabolic uptake and early detection of lesions [20, 21]. However, not all tumors have high radiotracer uptake, such as bronchoalveolar carcinoma, neuroendocrine tumors, colon mucinous adenocarcinoma, prostate cancer, carcinoids and so on [22]. In addition, some inflammatory lesions such as abscess, granulomatous disease, atherosclerosis, or benign tumors such as colon adenoma, uterine fibroids also have poor tracer uptake [23, 24].

In this study, we conducted a systematic review and meta-analysis on the diagnostic value of MRI and ^18^F-FDG PET/CT in ovarian cancer and indirectly compared the differential diagnosis performance of MRI and ^18^F-FDG PET/CT in ovarian benign and malignant tumors.

## Material and methods

### Study search strategy

This systematic review and meta-analysis were performed in accordance with PRISMA 2009 guidelines [25]. The Pubmed and Embase databases were searched for articles reporting on MRI or ^18^F-FDG PET/CT in ovarian cancer that were then included in the study. The following search terms were used: (“PET/CT” OR “PET-CT” OR “positron emission tomography/computed tomography” OR “positron emission tomography-computed tomography” OR “MR” OR “Magnetic Resonance”) AND (“ovarian cancer “OR “ovarian tumor” OR “ovarian neoplasms” OR “adnexal mass” OR “adnexal lesions”. Articles published in English language between January 2000 and January 2021 were included.

Two independent reviewers examined all potentially suitable articles after reading the abstract. When the results of two independent reviewers were inconsistent, a group discussion was held until a consensus was reached.

### Study selection

The studies needed to meet the following inclusion criteria: (i) published between January 2000 and January 2021; (ii) prospective or retrospective studies that evaluated the accuracy of ^18^F-FDG PET/CT or/and MRI in differentiating benign and malignant ovarian or adnexal tumors; (iii) reference standards that at least included histopathological examination results; (iv) research data that included or that allowed to derive true positive, false positive, false negative, and true negative values based on the sensitivity, specificity, accuracy, etc. provided in the article to construct a 2 × 2 contingency table. The exclusion criteria were: (i) the sample in the study was less than 10 patients; (ii) for MRI research, the magnetic field strength was < 1.5 T or the magnetic field strength information was not recorded; (iii) for PET/CT studies, other radiotracers were used; (iv) studies in which data or data subsets were published more than once.

### Data extraction and quality assessment

This meta-analysis extracted the first author, publication time, country, sample size, average age, study design type, patient selection (consecutive or nonconsecutive), true-positive (TP), false-positive (FP), false-negative (FN), true-negative (TN) results from the included studies. Other extracted data included: CT technology for PET/CT, magnetic field strength for MRI, the interval between index tests and HP, positive reference standard, the cutoff value of SUVmax for PET/CT, and ADC value for MRI of differentiating benign and malignant ovarian tumors. The Quality Assessment of Diagnostic Accuracy Studies-2 (QUADAS-2) was used for quality assessment of enrolled studies [26]. Data extraction and critical evaluation were independently carried out by two authors, if consensus could not be reached, a third reviewer was included to resolve disputes.

### Statistical analyses

Stata software version 14.0 (Stata Corporation, College Station, TX, USA) was used for the statistical processing of this meta-analysis, and *p* < 0.05 was considered to be statistically significant. The sensitivity, specificity, positive likelihood ratio (PLR), negative likelihood ratio (NLR), diagnostic odds ratio (DOR), and the area under the receiver operating characteristic (ROC) curves (AUC) with their 95% confidence intervals (CIs) for each individual study were calculated, according to the TP, FP, FN and TN values extracted from the enrolled study. The hierarchical logistic regression model was used to calculate general estimates of the sensitivity and specificity of the enrolled study, including the hierarchical summary receiver operating Characteristics (HSROC) model and concomitant variables. HSROC curves with 95% confidence and prediction regions were used to map the results for their sensitivity and specificity. PLR, NLR, and DOR were calculated by bivariate generalized linear mixed model and the random-effects model. Cochran’s Q test and Higgins I^2^ test were used to examine their heterogeneity [27]. In Cochran’s Q test, *p* < 0.05 was the test standard, indicating the existence of heterogeneity. Higgins I^2^ test was used to evaluate the degree of heterogeneity using the following criteria: inconsistency index (I^2^) < 50% was considered as irrelevant heterogeneity; I^2^ = 50–80% was deemed as the possibility of moderate heterogeneity; I^2^ > 80% suggested the possibility of significant heterogeneity. Two-sided *p* < 0.05 were considered as statistical significance across the included studies. The subgroup analysis of MRI and ^18^F-FDG PET/CT was carried out according to the sample size of the study, average age, study design type, patient selection, etc. The funnel plots and Deeks’ asymmetry tests were used as the assessment of publication bias for MRI and ^18^F-FDG PET/CT [28].

## Results

### Literature search

**Fig. 1:**
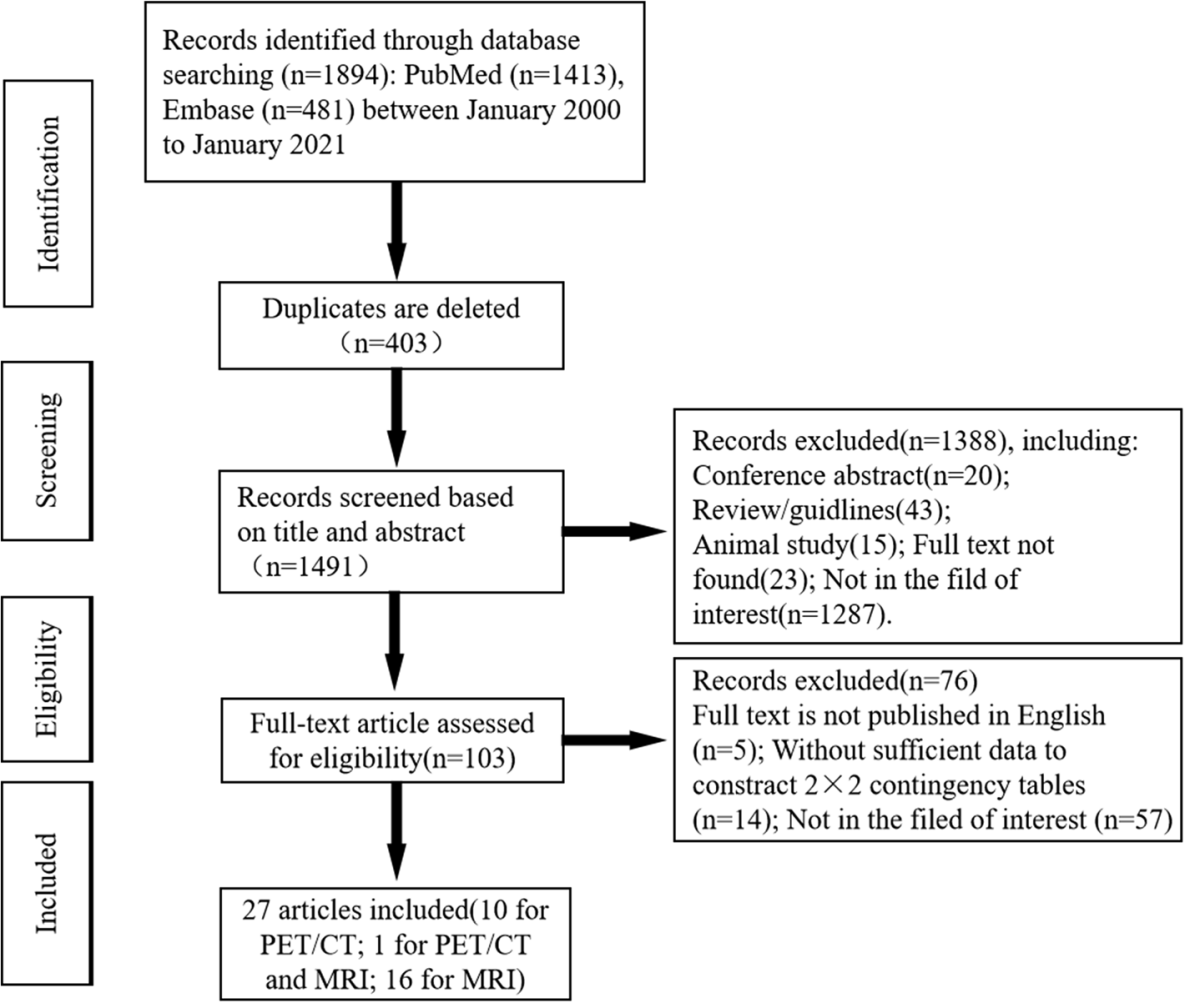
Flow chart of the research selection process

The literature search of related subject terms initially produced 1894 articles, consisting of 1413 articles in PubMed and 481 articles in Embase. After gradually deleting overlapping, irrelevant comments, case reports/series, conferences, animal research, studies that no provided a full text, and articles not in the field of interest, 1791 articles were excluded, and the remaining 103 potentially eligible original texts were further evaluated. As not all of them were completely published in English (*n* = 5), it was not possible to extract sufficient data to construct a 2 × 2 contingency table (*n* = 14) and further exclude papers in areas of non-interest (*n* = 57). Finally, 27 papers on the differentiation of benign and malignant ovarian or adnexal tumors were included for meta-analysis [29–55]. The detailed process of document retrieval is shown in Fig. 1.

### Study characteristics

The included 27 articles included 3730 patients with 3842 tumors, consisting of 10 PET/CT, 16 MRI, and 1 article that included PET/CT and MRI to differentiate benign and malignant ovarian or adnexal tumors. Among them, 17 studies were prospectively designed, 9 were retrospective studies, and 1 was unspecified. The sample size of the enrolled studies ranged from 30 to 1130 patients, and the average age of the patients ranged from 39.9 to 64.0 years old. For PET/CT, 7 out of the 11 studies recorded the cutoff value of the maximum standard uptake value (SUVmax) between benign and malignant tumors to study its diagnostic performance. As for MRI, 5 out of the 17 studies used the cutoff value of the apparent diffusion coefficient (ADC) value to distinguish benign and malignant ovarian tumors. In all the enrolled studies, histopathological examination was used as the reference standard, and 5 of them also included the follow-up time of at least half a year into the reference standard. The detailed characteristics of the enrolled studies and patients are summarized in Table 1**,** and the characteristics of PET/CT are shown in Table S1. The characteristics of MRI are shown in Table S2.

**Table 1:** The principal characteristics of eligible studies

Study /Year /Country	No. of patients	Mean age	Study design	Consecutive	Scanner	Cutoff value		Reference standard	Interval between index tests and HP	TP	FP	FN	FP
						SUVmax	ADC(10^−3^ mm^2^/s)						
Castellucci P /2007 /Italy [29]	50	64	P	Yes	PET/CT (Non-CE)	3.0		HP + follow-up	≤ 2 W	28	0	4	18
Kitajima K/2011 /Japan [30]	108(111tumor)	55.4	NR	NR	PET/CT (Non-CE + CE)	2.55		HP	NR	70	6	15	20
Zytoon AA/2012 /Egypt [31]	98	57.7	P	yes	PET/CT (Non-CE)	4.3		HP + follow-up	≤ 4 W	87	0	7	4
Risum S/2007 /Denmark [32]	97	60	P	yes	PET/CT (Non-CE + CE)	NR		HP	≤ 2 W	57	3	0	37
Tanizaki Y/2014 /Japan [33]	160	NR	R	NR	PET/CT (Non-CE)	2.9		HP	NR	54	5	13	88
Dauwen H/2013 /Belgium [34]	69	60	P	Yes	PET/CT (Non-CE + CE)	NR		HP	≤17d	52	3	4	10
Yamamoto Y/ 2008/Japan [35]	30	47.7	P	Yes	PET/CT (Non-CE)	3.0		HP	NR	10	3	4	13
Takagi H/2018 /Japan [36]	76	59	R	NR	PET/CT (Non-CE)	3.97		HP	NR	39	5	2	30
Michielsen K/2013 /Belgium [37]	32	61.9	P	Yes	PET/CT (Non-CE)	NR		HP	NR	29	2	0	1
Lee JW/2015/ Korea [38]	39	51	R	NR	PET/CT (Non-CE + CE)	2.5		HP	≤ 7 W	18	4	0	17
Nam EJ/2009 /Korea [39]	133	51	P	NR	PET/CT (Non-CE + CE)	NR		HP	≤ 4 W	93	10	2	28
Nam EJ/2009 /Korea [39]	133	51	P	NR	MRI (1.5 T)		NR	HP	≤ 4 W	69	7	3	6
Kawahara K/ 2004/Japan [40]	38	55.3	P	yes	MRI (1.5 T)		NR	HP	≤ 2 W	21	2	2	13
Kierans AS/2013 /USA [41]	37	54	R	Yes	MRI (1.5/3 T)		NR	HP	≤ 137D	6	3	3	25
Türkoğlu S/ 2020/Turkey [42]	43	51.26	R	Yes	MRI (1.5 T)		0.93	HP	≤ 1 W	15	5	8	15
Michielsen K/ 2017/Belgium [43]	161	NR	P	Yes	MRI (3 T)		NR	HP	NR	122	8	4	27
Uehara T/2012 /Japan [44]	50	51	R	Yes	MRI (3 T)		NR	HP	NR	18	3	1	28
Booth SJ/ 2008/UK [45]	191	56	R	NR	MRI (3 T)		NR	HP	NR	90	22	8	71
Shimada K/2017 /Japan [46]	265	NR	P	Yes	MRI (1.5 T)		NR	HP	≤4 M	52	18	2	193
Zhang H/2019 /China [47]	85	52.7	R	Yes	MRI(1.5 T)		1.162	HP	NR	51	3	11	20
Zhang P/2012/ China [48]	191(202tumor)	56.5	R	Yes	MRI (1.5 T)		1.20	HP	NR	85	43	7	67
Li W/2011/ China [49]	127(131tumor)	B = 46.2,M = 59.9	R	Yes	MRI (1.5 T)		1.25	HP	NR	77	5	8	41
Fan X/2015/ China [50]	64	46.7	R	NR	MRI (3 T)		0.878	HP	NR	54	5	4	25
Sohaib SA/2003/ UK [51]	104(155tumor)	50	P	Yes	MRI (1.5 T)		NR	HP	NR	61	11	3	80
Gity M/2019/ Iran [52]	43(49tumor)	39.9	P	Yes	MRI (3 T)		NR	HP	NR	21	9	1	18
Pereira PN/2018/Brazil [53]	200(237tumor)	B = 47.1;M = 57.8	P	Yes	MRI (1.5 T)		NR	HP + follow-up	NR	75	4	4	154
Van TP/2007/ UK [54]	76	B = 46;M = 57	P	Yes	MRI (1.5 T)		NR	HP	NR	23	7	2	44
Thomassin NI/ 2020/France [55]	1130	49	P	Yes	MRI (1.5/3 T)		NR	HP + follow-up	NR	189	79	14	848

*B* Belign, *M* Malignant, *HP* Histopathology, *P* Prospective, *R* Retrospective, *NR* Not report, *W* Week, *M* Month, *D* Day, *MRI* Magnetic resonance imaging, *PET/CT* Positron emission computer/Computed tomography, *T* Tesla

### Quality assessment

The quality of all studies was considered satisfactory if it met at least 5 out of the 7 reference standards (7 reference standards include four items in the risk of bias, patient selection, index test, reference standard, flow and timing and three in application concerns, patient selection, index test, reference standard). Regarding the risk of bias for reference standards, all studies included at least histopathological examinations, which are considered low-risk. Since most studies did not report the time interval between the index and reference standard tests, the risk of bias in flow and time was not assessed. Also, in two studies, the longtime interval between the index test and the reference standard test (within 4 months and 137 days, respectively) was considered a higher risk [41, 46]. In terms of patient selection, all patients included in this study were suspected of having ovarian tumors detected by ultrasound or serum tumor markers, and the risks of publication bias and application concerns were considered low. The results of the QUADAS-2 assessment are shown in Table S3.

### Diagnostic accuracy

**Fig. 2:**
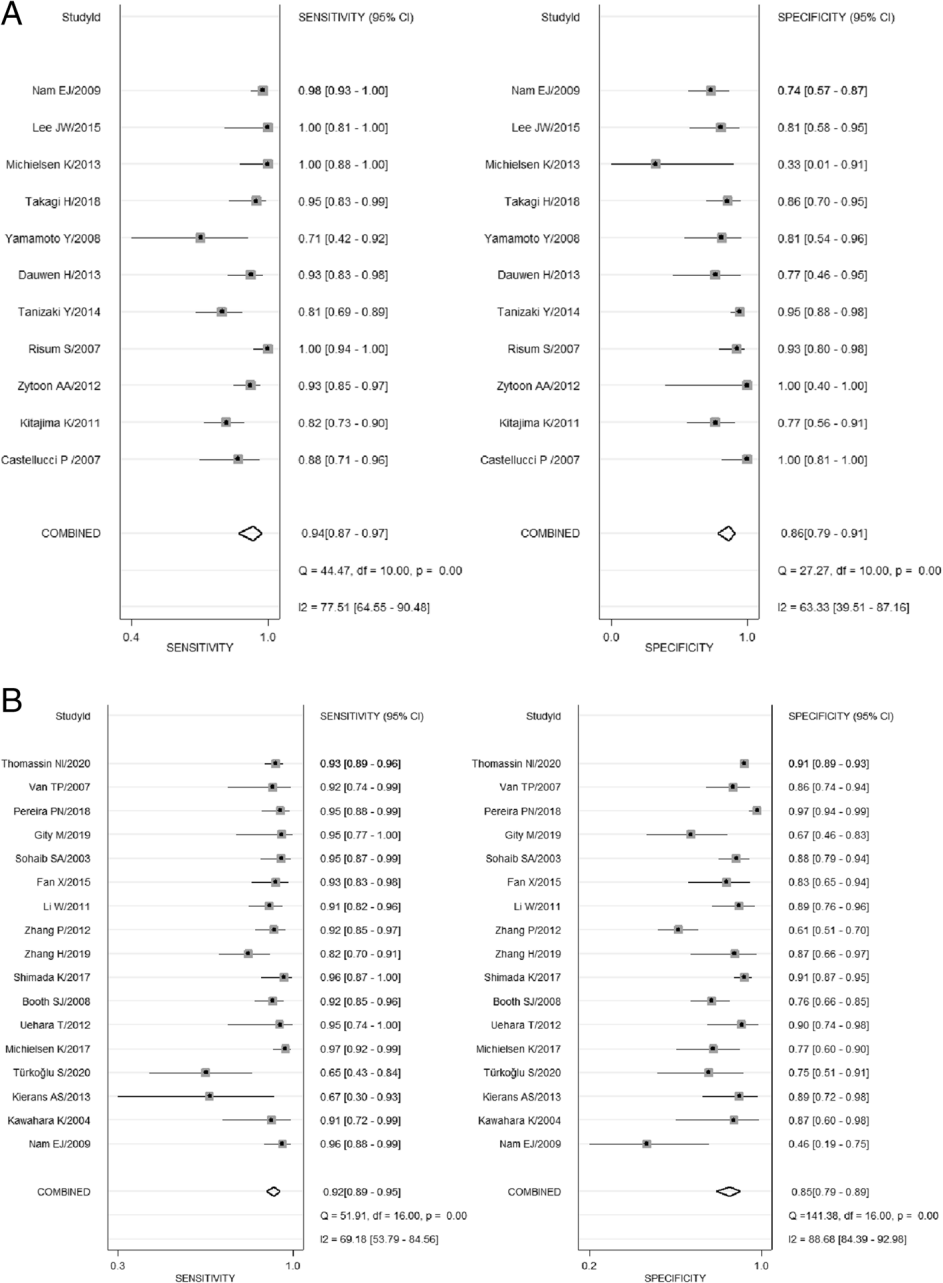
Forest plot of sensitivity and specificity of PET/CT(**A**) and MRI(**B**) in the diagnosis of ovarian cancer

The sensitivity of 11 studies containing ^18^F-FDG PET/CT methods to differentiate benign and malignant ovarian tumors ranged from 0.71 (95% CI, 0.42–0.92) to 1.0 (95% CI, 0.94–1.00), and the specificity was 0.33 (95% CI, 0.01–0.91) to 1.0 (95%CI, 0.81–1.00). Among them, a study that combined PET/CT with a Risk of Malignancy Index (RMI) based on serum CA-125, ultrasound examinations, and menopausal state showed high diagnostic value in differentiating benign and malignant ovarian tumors, with sensitivity and specificity of 1.0 (95%CI:0.94–1.0) and 0.93 (95%CI:0.80–0.98), respectively [32]. The pooled sensitivity and specificity of ^18^F-FDG PET/CT in differentiating benign and malignant ovarian tumors is 0.94 (95% CI, 0.87–0.97) and 0.86 (95% CI, 0.79–0.91), respectively, as shown in Fig. 2A. Both Cochran’s Q test and Higgins I^2^ test showed significant heterogeneity in sensitivity (Q = 42.89, *p* ≤ 0.01; I^2^ = 74.35) and specificity (Q = 19.65, p<0.01; I^2^ = 70.00).

A total of 17 studies included the diagnostic performance of MRI in diagnosing ovarian cancer, with sensitivity and specificity ranging from 0.65 (95% CI: 0.43–0.84) to 0.97 (95% CI: 0.92–0.99), and 0.46 (95% CI: 0.19–0.75) to 0.97 (95% CI: 0.94–0.99); the combined sensitivity and specificity were 0.92 (95% CI: 0.89–0.95) and 0.85 (95% CI: 0.79–0.89), respectively, as shown in Fig. 2B**.** Among these studies, a study based on the Ovarian-Adnexal Reporting Data System Magnetic Resonance Imaging (O-RADSMRI) score confirmed that the method could be used for risk stratification of ultrasound uncertain ovarian-adnexal masses and demonstrated high diagnostic performance [55]. Moreover, a study conducted by Zhang et al. concluded that the radiomic features extracted from MRI are highly correlated with the diagnostic accuracy, classification, and patient prognosis of ovarian cancer [47]. Also, Cochran’s Q test and Higgins I^2^ test showed heterogeneity between studies in sensitivity (Q = 151.02, *p* ≤ 0.01; I^2^ = 85.46) and specificity (Q = 54.27, *p* ≤ 0.01; I^2^ = 70.52).

**Fig. 3:**
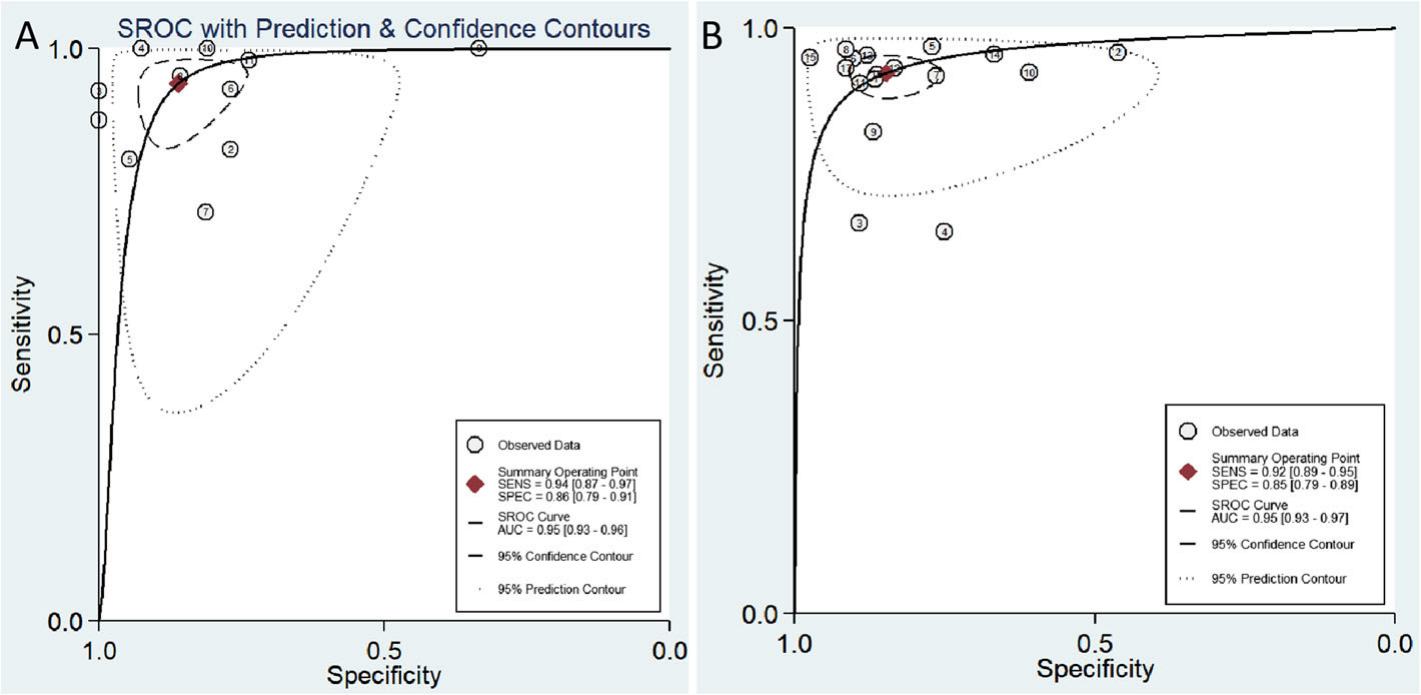
SROC curve of the diagnostic performance of FDG PET/CT(**A**) and (**B**) for ovarian cancer. AUC = area under the curve; SENS = sensitivity; SPEC = specificity; SROC = summary receiver operating characteristic

The pooled PLR and NLR of ^18^F-FDG PET/CT was 6.7 (95% CI: 4.3–10.4) and 0.07 (95% CI: 0.03–0.15), respectively. As for MRI, the combined effect estimates of PLR and NLR were 6.06 (95% CI: 4.24–8.66) and 0.09 (95% CI: 0.06–0.13), respectively. The combined DOR value of ovarian tumors diagnosed by ^18^F-FDG PET/CT was 95 (95% CI: 41–218), and the combined DOR value for MRI was 67 (95% CI: 38–118), respectively, as shown in Table 2. There was no statistical difference between the diagnostic odds ratio of MRI compared with that of PET/CT (*p* = 0.81). The area under the SROC curve of ^18^F-FDG PET/CT was 0.95, with a 0.93–0.96 of 95%CI. The difference between the 95% confidence contour and the prediction contour was significant, which also indicated the heterogeneity among studies. As for MRI, the area under the SROC curve was 0.95 (95%CI: 0.93–0.97), as shown in Fig. 3.

**Table 2:** Summary of the diagnostic performance characteristics of PET/CT and MRI in distinguishing benign and malignant ovarian tumors

Parameter	^18^F-FDG PET/CT		MRI	
	Estimate	95% CI	Estimate	95% CI
Sensitivity	0.94	0.87, 0.97	0.92	0.89, 0.95
Specificity	0.86	0.79, 0.91	0.85	0.79, 0.89
Positive Likelihood Ratio	6.7	4.3, 10.4	6.1	4.2, 8.7
Negative Likelihood Ratio	0.07	0.03, 0.15	0.09	0.06, 0.13
Diagnostic Odds Ratio	95	41, 218	67	38, 118
AUC	0.95	0.93, 0.96	0.95	0.93, 0.97

^*18*^*F-FDG* Fluorine-18 labeled deoxyglucose, *PET* Positron emission computer, *CT* Computed tomography, *MRI* Magnetic resonance imaging, *DWI* Diffusion weighted imaging, *CI* Confidence interval, *AUC* Area under curve

### Publication bias

**Fig. 4:**
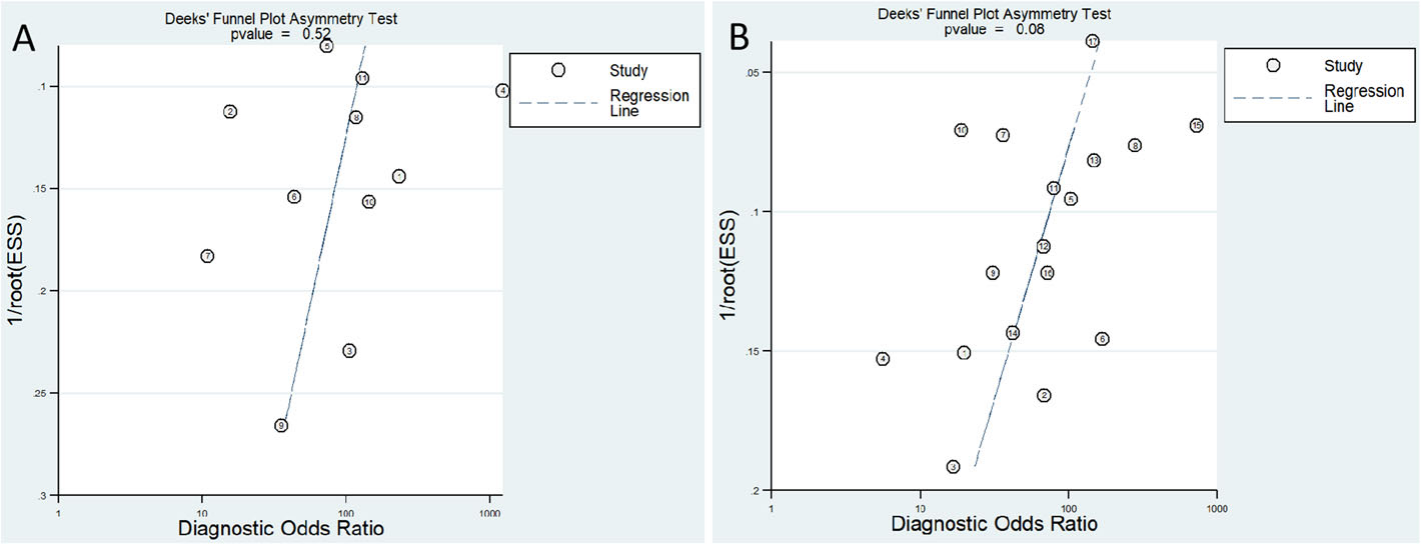
Deeks et al.’s funnel plot for publication bias for ^18^F-FDG PET/CT(**A**) and MRI(**B**)

Deeks et al.’s funnel plot for publication bias for MRI and ^18^F-FDG PET/CT is shown in Fig. 4. The *p* values of the slope coefficients were all greater than 0.05 (for PET/CT, *p* = 0.52, for MRI, *p* = 0.08), indicating that the possibility of publication bias between studies was low.

### Exploration of heterogeneity

The results of the meta regression analysis are summarized in Table 3. For both ^18^F-FDG PET/CT and MRI studies in the differentiation of benign and malignant ovarian tumors, the results of meta-regression analysis showed that the type of study design (prospective vs. retrospective) was a factor affecting heterogeneity (p<0.01). Specifically, for PET/CT, the sensitivity of prospective research design was higher than in retrospective research (0.95[95%CI:0.91–0.99] versus 0.94 [95%CI:0.85–1.00]), but the specificity was lower than retrospective research (0.86 [95%CI:0.77–0.94] versus 0.89 [95%CI:0.81–0.98]). As for MRI, the sensitivity and specificity of studies designed for prospective studies were higher than those in retrospective studies, which were 0.95 (95%CI:[0.93–0.97]) versus 0.88 (95%CI:[0.85–0.92]), 0.86 (95%CI: [0.80–0.93]) versus 0.83 (95%CI:[0.75–0.91]), respectively.

**Table 3:** The results of meta-regression analysis of PET/CT and MRI to diferenciate benign and malignant ovarian tumors

Covariates	Subgroup	No. of studies	Sensitivity(95%CI)	Specitivity(95%CI)	*P*
^18^F-FDG PET/CT					
Study design	Prospective	7	0.95 [0.91–0.99]	0.86 [0.77–0.94]	<0.01
	Retrospective	3	0.94 [0.85–1.00]	0.89 [0.81–0.98]	
CT technique	Without enhanced CT	5	0.96 [0.92–1.00]	0.81 [0.73–0.90]	0.21
	With enhanced CT	6	0.91 [0.83–0.99]	0.91 [0.85–0.96]	
Sample size	>50	7	0.94 [0.89–0.99]	0.87 [0.80–0.94]	0.93
	≤50	4	0.94 [0.86–1.00]	0.84 [0.71–0.97]	
Mean age	≥60	4	0.97 [0.93–1.00]	0.89 [0.82–0.96]	<0.01
	<60	6	0.93 [0.87–0.99]	0.80 [0.73–0.87]	
MRI^a^					
Study design	Prospective	9	0.95 [0.93–0.97]	0.86 [0.80–0.93]	<0.01
	Retrospective	8	0.88 [0.85–0.92]	0.83 [0.75–0.91]	
Magnetic field strength	With 1.5 T	12	0.91 [0.88–0.94]	0.86 [0.81–0.92]	0.22
	Only 3.0 T	5	0.95 [0.91–0.98]	0.80 [0.68–0.92]	
Scan sequence	With DWI	11	0.91 [0.88–0.95]	0.87 [0.82–0.93]	0.16
	Without DWI	6	0.94 [0.91–0.98]	0.78 [0.67–0.90]	
Sample size	>50	12	0.93 [0.91–0.96]	0.85 [0.79–0.91]	0.09
	≤50	5	0.85 [0.76–0.94]	0.83 [0.72–0.95]	
No. of imaging planes	3	7	0.92 [0.92–0.92]	0.83 [0.83–0.83]	1.00
	2	9	0.93 [0.93–0.93]	0.85 [0.85–0.85]	

^*18*^*F-FDG* Fluorine-18 labeled deoxyglucose, *PET* Positron emission computer, *CT* Computed tomography, *MRI* Magnetic resonance imaging, *DWI* Diffusion weighted imaging, *CI* Confidence interval; ^a^As for MRI, the mean age of all patients with ovarian tumors enrolled in the study was less than 60 years old, so they were not included in the subgroup analysis

In addition to the study design, the average age of patients between studies using PET/CT to differentiate benign and malignant ovarian tumors also showed heterogeneity. The sensitivity and specificity of the study with the average age of the enrolled patients older than 60 years old were higher than those in the group with the average age of enrolled patients younger than 60 years old, which were 0.97 (95%CI: [0.93–1.00]) versus 0.93 (95%CI: [0.87–0.99]) and 0.89(95%CI: [0.82–0.96]) versus 0.80(95%CI:[0.73–0.87]), respectively, and the difference was statistically significant (p<0.01). Otherwise, neither the sample size, nor the use of CT enhancement technology affected the heterogeneity between PET/CT studies, with *p* values of 0.93 and 0.21, respectively.

In addition, in terms of the magnetic field strength of MRI, the sensitivity of using 3.0 T was higher than 1.5 T (0.95 [95%CI:0.91–0.98] vs 0.91 [95%CI:0.88–0.94]), but the specificity was lower (0.80 [95%CI:0.68–0.92] vs 0.86 [95%CI:0.81–0.92]). However, the difference was not statistically significant (*p* = 0.22). Also, the number of imaging planes (2 or 3) was not a factor affecting the accuracy of MRI diagnosis (*p* = 1.0).

## Discussion

The current study evaluated the diagnostic performance of MRI and ^18^F-FDG PET/CT in differentiating benign and malignant ovarian or adnexal tumors. Our results showed that MRI and PET/CT both had high and similar sensitivity and specificity in the diagnosis of ovarian cancer. All studies included patients at risk; some studies also included patients confirmed with ovarian or appendage masses through ultrasound examination or elevated serum marker CA125. It should be pointed out that the study of Lee et al [38] contains a large number of ovarian lesions from other tumor sources, so we excluded the data of patients with ovarian metastases when performing this meta-analysis.

^18^F-FDG PET/CT and MRI data showed significant heterogeneity in the pooled sensitivity and specificity results. According to the results of meta-regression analysis, the statistically significant factors that caused the heterogeneity between PET/CT studies may be attributed to the type of study design and the average age of the enrolled patients. Specifically, retrospective studies showed lower sensitivity and higher specificity than prospective studies, which may be related to the small number of retrospective studies [33, 36, 38]. Moreover, the sensitivity and specificity were higher when examining patients older than 60 years old compared to studies that included patients younger than 60 years old; yet, the reason for this remains unclear. As for MRI, similar conclusions were drawn, i.e., the sensitivity and specificity of prospective design research were higher than retrospective research. When performing retrospective studies, doctors cannot always obtain enough clinical data, which may result in lower sensitivity and specificity.

Our meta-regression analysis showed that the use of enhanced CT technology and low-dose CT was not a heterogeneous factor affecting diagnostic performance. Therefore, from the perspective of patients receiving radiation doses, the use of enhanced CT technology needs to be reconsidered in future research. Also, as for MRI, further studies may be needed to truly determine the added value of DWI sequences in identifying benign and malignant ovarian tumors because meta-regression analysis showed no statistical difference between using DWI sequences and not using DWI sequences. It is worth noting that in both PET/CT and MRI studies, only two studies included follow-up as the reference standard. Therefore, studies using only pathological biopsy and combined pathological biopsy and follow-up time as reference standards were not included in meta-regression analysis.

The results of previous meta-analysis studies have shown that PET/CT has good diagnostic performance in ovarian cancer distant metastasis and prognostic evaluation [56–58]. Specifically, the meta-analysis results of Han et al. showed that the pooled sensitivity and specificity of ^18^F-FDG PET/CT in identifying distant metastases of ovarian cancer was 0.72 (95% CI: 0.61–0.81) and 0.93 (95% CI: 0.85–0.97), respectively. Among them, the pooled sensitivity and specificity of PET/CT in the diagnosis of retroperitoneal lymph node metastasis was 0.77 (95%CI: 0.61–0.87) and 0.97 (95%CI: 0.93–0.99), respectively [56]. Meanwhile, another study by Han et al. showed that ^18^F-FDG-PET/CT-derived volume-based metabolic parameters were statistically significant prognostic factors in terms of progression-free (PFS) and overall survival (OS) in patients with ovarian cancer. Patients with a high MTV or TLG were at higher risk of disease progression or death [57]. Moreover, in their meta-analysis of 64 studies with 3722 patients, Xu et al. showed that the pooled sensitivity and specificity of PET/CT and PET for recurrent/metastatic ovarian cancer were 0.92 (95%CI: 0.90—0.93) and 0.91(95%CI: 0.89–0.93), respectively [58]. Our meta-analysis included 11 PET/CT studies that differentiated benign and malignant ovarian or adnexal tumors with a good diagnostic performance of PET/CT in ovarian cancer [29–39]. However, the main purpose of our study was to compare the diagnostic performance of PET/CT and MRI in differentiating benign and malignant primary ovarian tumors. Our results showed that both methods had good diagnostic performance; thus, both methods should be recommended in clinical practice. Compared with PET/CT, MRI can shorten the examination time and lower medical costs [59]. E.g., if we consider GE or Siemens MRI with a magnetic field strength of 1.5 T; when doing lower abdomen and pelvic examinations, the scanning time of conventional T1WI, T2WI sequence plus DWI sequence is less than 10 min; the time for a PET/CT examination is often more than 15 min. In China, the cost of an MRI is significantly lower than that of PET/CT. Also, MRI does not produce ionizing radiation and is commonly recommended for female patients (protecting breasts and ovaries sensitive to radiation) [60]. On the other hand, PET/CT has good diagnostic performance in identifying benign and malignant ovarian tumors. PET/CT may also detect ovarian cancer metastases at the same time. PET/CT is recommended for ovarian cancer staging and treatment evaluation. Therefore, future prospective studies comparing whole-body MRI and PET/CT in the staging of ovarian cancer are warranted. Since the current study only discussed the diagnostic value of MRI and PET/CT in distinguishing benign and malignant ovarian tumors, based on the above advantages of MRI, we believe that MRI may be more suitable as an auxiliary examination method for the differentiation of benign and malignant ovarian tumors.

The main limitation of the present study was that it indirectly compared the diagnostic accuracy of PET/CT and MRI. Different methods and different characteristics of patients were included in those studies, resulting in great heterogeneity in the estimation of diagnostic accuracy, limiting the quality of this meta-analysis. Secondly, in both PET/CT and MRI studies, inconsistent interpretation of the results was also a major drawback. For instance, some studies classified borderline ovarian tumors as benign tumors [31, 32], but some studies classified them as malignant tumors [30, 33, 35, 39, 46, 52, 54]. Moreover, most of the studies included in the analysis did not describe the quantitative data of ovarian tumors, such as the average size of benign and malignant tumors, the SUVmax value for the PET/CT study, the ADC value for the MRI study, which limits further subgroup analysis research. Nonetheless, the study with a large sample size indirectly compared MRI with ^18^F-FDG PET/CT still provides a reference for the differential diagnosis of benign and malignant ovarian or adnexal tumors.

## Conclusion

MRI and ^18^F-FDG PET/CT showed to have high and similar diagnostic performance in the differential diagnosis of benign and malignant ovarian or adnexal tumors. MRI is a promising non-radiation imaging technology, which may be a more favorable choice for patients with ovarian or accessory tumors. Prospective studies directly comparing MRI and 18F-FDG PET/CT diagnostic performance in the differentiation of benign and malignant ovarian or adnexal tumors are needed in the future.

## Supplementary Information

**Additional file 1 Table S1**. FDG PET/CT characteristics. **Table S2**. MRI characteristics. **Table S3**. Risk of bias and application concerns for included studies were assessed by the QUADAS-2 tool.

## Data Availability

The datasets used and materials during the current study are available from the corresponding author on reasonable request.

## References

[1] Bray F, Ferlay J, Soerjomataram I, Siegel RL, Torre LA, Jemal A (2018). Global cancer statistics 2018: GLOBOCAN estimates of incidence and mortality worldwide for 36 cancers in 185 countries. CA Cancer J Clin.

[2] Kurman RJ, Shih IM (2016). The dualistic model of ovarian carcinogenesis: revisited, revised, and expanded. Am J Pathol.

[3] Bowtell DD (2010). The genesis and evolution of high-grade serous ovarian cancer. Nat Rev Cancer.

[4] Adami HO, Hsieh CC, Lambe M, Trichopoulos D, Leon D, Persson I, Ekbom A, Janson PO (1994). Parity, age at first childbirth, and risk of ovarian cancer. Lancet.

[5] Ziogas A, Gildea M, Cohen P, Bringman D, Taylor TH, Seminara D, Barker D, Casey G, Haile R, Liao SY, Thomas D, Noble B, Kurosaki T, Anton-Culver H (2000). Cancer risk estimates for family members of a population-based family registry for breast and ovarian cancer. Cancer Epidemiol Biomark Prev.

[6] Gates MA, Rosner BA, Hecht JL, Tworoger SS (2010). Risk factors for epithelial ovarian cancer by histologic subtype. Am J Epidemiol.

[7] Braem MG, Onland-Moret NC, van den Brandt PA, Goldbohm RA, Peeters PH, Kruitwagen RF, Schouten LJ (2010). Reproductive and hormonal factors in association with ovarian cancer in the Netherlands cohort study. Am J Epidemiol.

[8] Moorman PG, Palmieri RT, Akushevich L, Berchuck A, Schildkraut JM (2009). Ovarian cancer risk factors in African-American and white women. Am J Epidemiol.

[9] Schouten LJ, Rivera C, Hunter DJ, Spiegelman D, Adami HO, Arslan A, Beeson WL, van den Brandt PA, Buring JE, Folsom AR, Fraser GE, Freudenheim JL, Goldbohm RA, Hankinson SE, Lacey JV, Leitzmann M, Lukanova A, Marshall JR, Miller AB, Patel AV, Rodriguez C, Rohan TE, Ross JA, Wolk A, Zhang SM, Smith-Warner SA (2008). Height, body mass index, and ovarian cancer a pooled analysis of 12 cohort studies. Cancer Epidemiol Biomarkers Prev.

[10] Ness RB, Dodge RC, Edwards RP, Baker JA, Moysich KB (2011). Contraception methods, beyond oral contraceptives and tubal ligation, and risk of ovarian cancer. Ann Epidemiol.

[11] Kotsopoulos J, Vitonis AF, Terry KL, De Vivo I, Cramer DW, Hankinson SE, Tworoger SS (2009). Coffee intake, variants in genes involv ed in caffeine metabolism and the risk of epithefial ovarian cancer. Cancer Causes Control.

[12] Mathieu KB, Bedi DG, Thrower SL, Qayyum A, Bast RC (2018). Screening for ovarian cancer: imaging challenges and opportunities for improvement. Ultrasound Obstet Gynecol.

[13] Terry KL, Schock H, Fortner RT, Hüsing A, Fichorova RN, Yamamoto HS, Vitonis AF, Johnson T, Overvad K, Tjønneland A, Boutron-Ruault MC, Mesrine S, Severi G, Dossus L, Rinaldi S, Boeing H, Benetou V, Lagiou P, Trichopoulou A, Krogh V, Kuhn E, Panico S, Bueno-de-Mesquita HB, Onland-Moret NC, Peeters PH, Gram IT, Weiderpass E, Duell EJ, Sanchez MJ, Ardanaz E, Etxezarreta N, Navarro C, Idahl A, Lundin E, Jirström K, Manjer J, Wareham NJ, Khaw KT, Byrne KS, Travis RC, Gunter MJ, Merritt MA, Riboli E, Cramer DW, Kaaks R (2016). A Prospective Evaluation of Early Detection Biomarkers for Ovarian Cancer in the European EPIC Cohort. Clin Cancer Res.

[14] Lheureux S, Gourley C, Vergote I, Oza AM (2019). Epithelial ovarian cancer. Lancet.

[15] Zhang M, Zhang Y, Fu J, Zhang L (2019). Serum CA125 levels are decreased in rectal cancer but increased in fibrosis-associated diseases and in most types of cancers. Prog Mol Biol Transl Sci.

[16] Chen F, Shen J, Wang J, Cai P, Huang Y (2018). Clinical analysis of four serum tumor markers in 458 patients with ovarian tumors: diagnostic value of the combined use of HE4, CA125, CA19-9, and CEA in ovarian tumors. Cancer Manag Res.

[17] Kobayashi E, Ueda Y, Matsuzaki S, Yokoyama T, Kimura T, Yoshino K, Fujita M, Kimura T, Enomoto T (2012). Biomarkers for screening, diagnosis, and monitoring of ovarian cancer. Cancer Epidemiol Biomark Prev.

[18] van Nagell JR, PD DP, Ueland FR, CP DS, Cooper AL, JM MD, Pavlik EJ, Kryscio RJ (2007). Ovarian cancer screening with annual transvaginal sonography: findings of 25,000 women screened. Cancer.

[19] Pykett IL, Newhouse JH, Buonanno FS, Brady TJ, Goldman MR, Kistler JP, Pohost GM (1982). Principles of nuclear magnetic resonance imaging. Radiology.

[20] Haubner R, Weber WA, Beer AJ, Vabuliene E, Reim D, Sarbia M, Becker KF, Goebel M, Hein R, Wester HJ, Kessler H, Schwaiger M (2005). Noninvasive visualization of the activated alphavbeta3 integrin in cancer patients by positron emission tomography and [18F]Galacto-RGD. PLoS Med.

[21] Basu S, Kwee TC, Surti S, Akin EA, Yoo D, Alavi A (2011). Fundamentals of PET and PET/CT imaging. Ann N Y Acad Sci.

[22] Manenti G, Cicciò C, Squillaci E, Strigari L, Calabria F, Danieli R, Schillaci O, Simonetti G (2012). Role of combined DWIBS/3D-CE-T1w whole-body MRI in tumor staging: comparison with PET-CT. Eur J Radiol.

[23] Kemppainen J, Hynninen J, Virtanen J, Seppänen M (2019). PET/CT for evaluation of ovarian Cancer. Semin Nucl Med.

[24] Blake MA, Singh A, Setty BN, Slattery J, Kalra M, Maher MM, Sahani DV, Fischman AJ, Mueller PR (2006). Pearls and pitfalls in interpretation of abdominal and pelvic PET-CT. Radiographics.

[25] Moher D, Liberati A, Tetzlaff J, Altman DG, PRISMA Group (2009). Preferred reporting items for systematic reviews and meta-analyses: the PRISMA statement. PLoS Med.

[26] Whiting PF, Rutjes AW, Westwood ME, Mallett S, Deeks JJ, Reitsma JB, Leeflang MM, Sterne JA, Bossuyt PM, QUADAS-2 group (2011). QUADAS-2: a revised tool for the quality assessment of diagnostic accuracy studies. Ann Intern Med.

[27] Higgins JP, Thompson SG, Deeks JJ, Altman DG (2003). Measuring inconsistency in meta-analyses. BMJ.

[28] Deeks JJ, Macaskill P, Irwig L (2005). The performance of tests of publication bias and other sample size effects in systematic reviews of diagnostic test accuracy was assessed. J Clin Epidemiol.

[29] Castellucci P, Perrone AM, Picchio M, Ghi T, Farsad M, Nanni C, Messa C, Meriggiola MC, Pelusi G, Al-Nahhas A, Rubello D, Fazio F, Fanti S (2007). Diagnostic accuracy of 18F-FDG PET/CT in characterizing ovarian lesions and staging ovarian cancer: correlation with transvaginal ultrasonography, computed tomography, and histology. Nucl Med Commun.

[30] Kitajima K, Suzuki K, Senda M, Kita M, Nakamoto Y, Onishi Y, Maeda T, Yoshikawa T, Ohno Y, Sugimura K (2011). FDG-PET/CT for diagnosis of primary ovarian cancer. Nucl Med Commun.

[31] Zytoon AA, Murakami K, Eid H, El-Gammal M (2013). High impact of FDG-PET/CT in diagnostic strategies for ovarian cancer. Acta Radiol.

[32] Risum S, Høgdall C, Loft A, Berthelsen AK, Høgdall E, Nedergaard L, Lundvall L, Engelholm SA (2007). The diagnostic value of PET/CT for primary ovarian cancer--a prospective study. Gynecol Oncol.

[33] Tanizaki Y, Kobayashi A, Shiro M, Ota N, Takano R, Mabuchi Y, Yagi S, Minami S, Terada M, Ino K (2014). Diagnostic value of preoperative SUVmax on FDG-PET/CT for the detection of ovarian cancer. Int J Gynecol Cancer.

[34] Dauwen H, Van Calster B, Deroose CM, Op de Beeck K, Amant F, Neven P, Berteloot P, Leunen K, Timmerman D, Vergote I (2013). PET/CT in the staging of patients with a pelvic mass suspicious for ovarian cancer. Gynecol Oncol.

[35] Yamamoto Y, Oguri H, Yamada R, Maeda N, Kohsaki S, Fukaya T (2008). Preoperative evaluation of pelvic masses with combined ^18^F-fluorodeoxyglucose positron emission tomography and computed tomography. Int J Gynaecol Obstet.

[36] Takagi H, Sakamoto J, Osaka Y, Shibata T, Fujita S, Sasagawa T (2018). Utility of ^18^F-fluorodeoxyglucose-positron emission tomography in the differential diagnosis of benign and malignant gynaecological tumours. J Med Imaging Radiat Oncol.

[37] Michielsen K, Vergote I, Op de Beeck K, Amant F, Leunen K, Moerman P, Deroose C, Souverijns G, Dymarkowski S, De Keyzer F, Vandecaveye V (2014). Whole-body MRI with diffusion-weighted sequence for staging of patients with suspected ovarian cancer: a clinical feasibility study in comparison to CT and FDG-PET/CT. Eur Radiol.

[38] Lee JW, Lee JH, Cho A, Yun M, Lee JD, Kim YT, Kang WJ (2015). The performance of contrast-enhanced FDG PET/CT for the differential diagnosis of unexpected ovarian mass lesions in patients with nongynecologic cancer. Clin Nucl Med.

[39] Nam EJ, Yun MJ, Oh YT, Kim JW, Kim JH, Kim S, Jung YW, Kim SW, Kim YT (2010). Diagnosis and staging of primary ovarian cancer: correlation between PET/CT, Doppler US, and CT or MRI. Gynecol Oncol.

[40] Kawahara K, Yoshida Y, Kurokawa T, Suzuki Y, Nagahara K, Tsuchida T, Okazawa H, Fujibayashi Y, Yonekura Y, Kotsuji F (2004). Evaluation of positron emission tomography with tracer 18-fluorodeoxyglucose in addition to magnetic resonance imaging in the diagnosis of ovarian cancer in selected women after ultrasonography. J Comput Assist Tomogr.

[41] Kierans AS, Bennett GL, Mussi TC, Babb JS, Rusinek H, Melamed J, Rosenkrantz AB (2013). Characterization of malignancy of adnexal lesions using ADC entropy: comparison with mean ADC and qualitative DWI assessment. J Magn Reson Imaging.

[42] Türkoğlu S, Kayan M (2020). Differentiation between benign and malignant ovarian masses using multiparametric MRI. Diagn Interv Imaging.

[43] Michielsen K, Dresen R, Vanslembrouck R, De Keyzer F, Amant F, Mussen E, Leunen K, Berteloot P, Moerman P, Vergote I, Vandecaveye V (2017). Diagnostic value of whole body diffusion-weighted MRI compared to computed tomography for pre-operative assessment of patients suspected for ovarian cancer. Eur J Cancer.

[44] Uehara T, Takahama J, Marugami N, Takahashi A, Takewa M, Itoh T, Kitano S, Nakagawa H, Kichikawa K (2012). Visualization of ovarian tumors using 3T MR imaging: diagnostic effectiveness and difficulties. Magn Reson Med Sci.

[45] Booth SJ, Turnbull LW, Poole DR, Richmond I (2008). The accurate staging of ovarian cancer using 3T magnetic resonance imaging--a realistic option. BJOG.

[46] Shimada K, Matsumoto K, Mimura T, Ishikawa T, Munechika J, Ohgiya Y, Kushima M, Hirose Y, Asami Y, Iitsuka C, Miyamoto S, Onuki M, Tsunoda H, Matsuoka R, Ichizuka K, Sekizawa A (2018). Ultrasound-based logistic regression model LR2 versus magnetic resonance imaging for discriminating between benign and malignant adnexal masses: a prospective study. Int J Clin Oncol.

[47] Zhang H, Mao Y, Chen X, Wu G, Liu X, Zhang P, Bai Y, Lu P, Yao W, Wang Y, Yu J, Zhang G (2019). Magnetic resonance imaging radiomics in categorizing ovarian masses and predicting clinical outcome: a preliminary study. Eur Radiol.

[48] Zhang P, Cui Y, Li W, Ren G, Chu C, Wu X (2012). Diagnostic accuracy of diffusion-weighted imaging with conventional MR imaging for differentiating complex solid and cystic ovarian tumors at 1.5T. World J Surg Oncol.

[49] Li W, Chu C, Cui Y, Zhang P, Zhu M (2012). Diffusion-weighted MRI: a useful technique to discriminate benign versus malignant ovarian surface epithelial tumors with solid and cystic components. Abdom Imaging.

[50] Fan X, Zhang H, Meng S, Zhang J, Zhang C (2015). Role of diffusion-weighted magnetic resonance imaging in differentiating malignancies from benign ovarian tumors. Int J Clin Exp Med.

[51] Sohaib SA, Sahdev A, Van Trappen P, Jacobs IJ, Reznek RH (2003). Characterization of adnexal mass lesions on MR imaging. AJR Am J Roentgenol.

[52] Gity M, Parviz S, Saligheh Rad H, Fathi Kazerooni A, Shirali E, Shakiba M, Baikpour M (2019). Differentiation of benign from malignant adnexal masses by dynamic contrast-enhanced MRI (DCE-MRI): quantitative and semi-quantitative analysis at 3-tesla MRI. Asian Pac J Cancer Prev.

[53] Pereira PN, Sarian LO, Yoshida A, Araújo KG, Barros RHO, Baião AC, Parente DB, Derchain S (2018). Accuracy of the ADNEX MR scoring system based on a simplified MRI protocol for the assessment of adnexal masses. Diagn Interv Radiol.

[54] Van TP, Rufford BD, Mills TD, Sohaib SA, Webb JA, Sahdev A, Carroll MJ, Britton KE, Reznek RH, Jacobs IJ (2007). Differential diagnosis of adnexal masses: risk of malignancy index, ultrasonography, magnetic resonance imaging, and radioimmunoscintigraphy. Int J Gynecol Cancer.

[55] Thomassin NI, Poncelet E, Jalaguier-Coudray A, Guerra A, Fournier LS, Stojanovic S, Millet I, Bharwani N, Juhan V, Cunha TM, Masselli G, Balleyguier C, Malhaire C, Perrot NF, Sadowski EA, Bazot M, Taourel P, Porcher R, Darai E, Reinhold C, Rockall AG (2020). Ovarian-adnexal reporting data system magnetic resonance imaging (O-RADS MRI) score for risk stratification of Sonographically indeterminate adnexal masses. JAMA Netw Open.

[56] Han S, Woo S, Suh CH, Lee JJ (2018). Performance of pre-treatment ^18^F-fluorodeoxyglucose positron emission tomography/computed tomography for detecting metastasis in ovarian cancer: a systematic review and meta-analysis. J Gynecol Oncol.

[57] Han S, Kim H, Kim YJ, Suh CH, Woo S (2018). Prognostic value of volume-based metabolic parameters of 18F-FDG PET/CT in ovarian cancer: a systematic review and meta-analysis. Ann Nucl Med.

[58] Xu B, Ma J, Jiang G, Wang Y, Ma Q (2017). Diagnostic value of positron emission tomography (PET) and PET/computed tomography in recurrent/metastatic ovarian cancer: a meta-analysis. J Obstet Gynaecol Res.

[59] Plathow C, Walz M, Lichy MP, Aschoff P, Pfannenberg C, Bock H, Eschmann SM, Claussen CD, Schlemmer HP (2008). Kostenüberlegungen zur Ganzkörper-MRT und PET-CT im Rahmen des onkologischen Stagings [cost considerations for whole-body MRI and PET/CT as part of oncologic staging]. Radiologe.

[60] Morone M, Bali MA, Tunariu N, Messiou C, Blackledge M, Grazioli L, Koh DM (2017). Whole-Body MRI: Current applications in oncology. AJR Am J Roentgenol.

