# Supplementary material for: Indirect comparison of the diagnostic performance of 18F-FDG PET/CT and MRI in differentiating benign and malignant ovarian or adnexal tumors: a systematic review and meta-analysis
Source: BMC Cancer. 2021 Oct 6;21:1080. doi: 10.1186/s12885-021-08815-3 (PMC8495994; doi:10.1186/s12885-021-08815-3)
Supplement: Supplementary file 1 — Additional file 1 Table S1. FDG PET/CT characteristics. Table S2. MRI characteristics. Table S3. Risk of bias and application concerns for included studies were assessed by the QUADAS-2 tool. [file 12885_2021_8815_MOESM1_ESM.doc]

| **Table S1. FDG PET/CT characteristics** | | | | | | | | | |
| --- | --- | --- | --- | --- | --- | --- | --- | --- | --- |
| Study | Vendor | machine | STmin（mm） | FDG dose | Time between FDG  administration  and scanning (min) | CT technique | Criteria for positivity | Interval between index test and HP | No. of readers |
| Castellucci P /2007 /Italy[29] | GE | Discovery LS | NR | 5.5MBq/kg | 60-90 | Non-CE,  4min/bed | SUVmax≥3.0. | NR | NR |
| Kitajima K/2011 /Japan[30] | GE | Discovery ST | 3.75 | 3.33MBq/kg | 50 | Non-CE=36  CE=72,  2min/bed | SUVmax≥2.55 | NR | NR |
| Zytoon AA/2012 /Egypt[31] | Siemens | Biograph Sensation 16 | 3.75 | 3.7-4.5MBq/kg | 40-60(Mean=50) | Non-CE,  2min/bed | NR | 16.3+10.4 | 2 |
| Risum S/2007 /Denmark[32] | GE | Discovery | 5 | 350-400MBq | 60 | Non-CE  CE | SUVmax≥4.3 | 14d | 1 |
| Tanizaki Y/2014 /Japan[33] | Shimadzu | SET-3000BCT/L | 2 | 2.6MBq/kg | 50 | Non-CE | SUVmax≥2.9 | NR | 2 |
| Dauwen H/2013 /Belgium[34] | Siemens | Biograph | - | 185-428MBq | 60-75 | Non-CE  CE/  3min/bed | NR | ≤17d | 1 |
| Yamamoto Y/ 2008/Japan[35] | GE | Discovery LS | 3.75 | 4.0MBq/kg | 50 | Non-CE;  2min/bed | SUVmax≥3.0 | NR | NR |
| Takagi H/2018 /Japan[36] | Siemens | Biograph Sensation 16 |  | 185MBq | 60 | Non-CE | SUVmax≥3.97 | NR | NR |
| Michielsen K/2013 /Belgium[37] | Siemens | Biograph 40 | 5 | Mean=303 | Mean=69 | Non-CE | NR |  | NR |
| Lee JW/2015/  Korea[38] | GE/Siemens | Discovery LS  /Biograph 40 | - | 5.5MBq/kg | 60 | Non-CE  CE/  3min/bed | SUVmax≥2.5 | 17±29days | 2 |
| Nam EJ/2009 /Korea[39] | Philips+GE | GEMIN/Discovery | 3 | 8-12mci | 60-90 | Non-CE  CE/  2min/bed | NR | ≤28d | 2 |

Abbreviations: GE=General Electric；STmin=minimum slice thickness；NR= not reported; CT=computed tomography; HP=histopathological; CE=Contrast enhancement; Non-CE=None contrast enhancement.

**Table S2. MRI characteristics**

| Study | Magnet strength | vendor | coverage | Seque-  nce used | No.of  imaging  planes | MST  (mm) | DWI | | T1WI | | T2WI | | CE-MR | No. of readers |
| --- | --- | --- | --- | --- | --- | --- | --- | --- | --- | --- | --- | --- | --- | --- |
|  |  |  |  |  |  |  | IP | ST(mm) | IP | ST  (mm) | IP | ST(mm) |  |  |
| Nam EJ/2009 /Korea[39] | 1.5T | Philips | ABD+pelvic | T1/T2 | 3 | 5 | - | - | A+S | 5 | A+S+C | 5 | Y | NR |
| Kawahara K/ 2004/Japan[40] | 1.5T | GE | pelvic | T1/T2 | 2 | 6 | - | - | A+S | 6 | A+S | 6 | Y | NR |
| Kierans AS/2013 /USA[41] | 1.5T | Siemens | pelvic | T1/T2/DWI | 3 | 6-8 | A+S | - | A+S | - | A+S | - | Y | 2 |
| Türkoğlu S/ 2020/Turkey[42] | 1.5T | Siemens | pelvic | T1/T2/DWI | 3 | 5 | A | 6 | A+C+S | 3 | A+C+S | 4 | Y | 2 |
| Michielsen K/ 2017/Belgium[43] | 3T | Philips | Whole body | T1/T2/DWI | 3 | 2.5 | A+C+S | 5 | A+C | 2.5 | C | 6 | Y | 2 |
| Uehara T/2012 /Japan[44] | 3T | Siemens | pelvic | T1/T2/DWI | 2 | - | A | 4.5 | A | 3 | A+S | 3 | Y | 2 |
| Booth SJ/ 2008/UK[45] | 3T | GE | pelvic | T1/T2 | 3 | - | - | - | A | - | A+S+oblique | - | Y | 1 |
| Shimada K/2017 /Japan[46] | 1.5T | Siemens | pelvic | T1/T2/DWI | 2 | 3.5-5 | A | - | A | - | A+S | - | Y | 2 |
| Zhang H/2019 /China[47] | 1.5T | Siemens | pelvic | T1/T2/DWI | 2 | 5 | A+C+S | 5 | A | 3 | A+C+S | 5 | Y | 2 |
| Zhang P/2012/ China[48] | 1.5T | GE | pelvic | T1/T2/DWI | 2 | 5 | A+C+S | 5 | A+S | 5 | A+S | 5 | Y | 2 |
| Li W/2011/ China[49] | 1.5T | GE | pelvic | T1/T2/DWI | 2 | 6 | A+S | 6 | A+S | 6 | A+S | 6 | Y | 2 |
| Fan X/2015/ China[50] | 3.0T | GE | ABD+pelvic | T1/T2/DWI | 3 | 5 | A+C+S | 5 | A+C+S | 5 | A+C+S | 5 | Y | 2 |
| Sohaib SA/2003/ UK[51] | 1.5T | GE | pelvic | T1/T2 | 2 | 5-8 | - | - | A | 5-8 | A+S | 5-7 | Y | 2 |
| Gity M/2019/ Iran[52] | 3T | Siemens | pelvic | T1/T2 | 2 | 5 | - | - | A+S | 5 | A+S | 5 | Y | 2 |
| Pereira PN/2018/Brazil[53] | 1.5T | GE | pelvic | T1/T2/DWI | 3 | 3.8 | A | 5 | A | 5 | A+C+S | 5 | Y | 2 |
| Van TP/2007/ UK[54] | 1.5T | GE | pelvic | T1/T2 | 2 | - | - | - | A | - | A+S | - | Y | 2 |
| Thomassin NI/ 2020/France[55] | 1.5/3.0T | Siemens/Philips/GE | pelvic | T1/T2/DWI | - | - | - | - | - | - | - | - | Y | 5 |

MST=minimum slice thickness; ST=slice thickness; A = axial; C = coronal; DCE = dynamic contrast enhanced; DWI = diffusion-weighted imaging; MRI = magnetic resonance imaging; ABD=abdoman; NR = not reported; T1WI = T1-weighted imaging; T2WI = T2-weighted imaging; S = sagittal; STIR = short tau inversion recovery，IP=Imaging plane.

| **Table S3. Risk of bias and application concerns for included studies were assessed by the QUADAS-2 tool.** | | | | | | | |
| --- | --- | --- | --- | --- | --- | --- | --- |
| Study | Risk of bias | | | | Application concerns | | |
|  | Patient selection | Index  test | Reference standard | Flow and  timing | Patient  selection | Index  test | Reference  standard |
| Castellucci P /2007 | L | U | L | L | L | L | L |
| Kitajima K/2011 | L | L | L | U | L | L | L |
| Zytoon AA/2012 | L | L | L | L | L | L | L |
| Risum S/2007 | L | L | L | L | L | U | L |
| Tanizaki Y/2014 | L | L | L | U | L | L | L |
| Dauwen H/2013 | L | L | L | L | L | L | L |
| Yamamoto Y/ 2008 | L | L | L | U | L | L | L |
| Takagi H/2018 | L | L | L | U | L | U | L |
| Michielsen K/2013 | L | L | L | U | L | L | L |
| Lee JW/2015 | L | U | L | L | L | L | L |
| Nam EJ/2009 | L | L | L | L | L | L | L |
| Kawahara K/ 2004 | L | L | L | L | L | L | L |
| Kierans AS/2013 | L | L | L | L | L | L | L |
| Türkoğlu S/ 2020 | L | U | L | H | L | L | L |
| Michielsen K/ 2017 | L | L | L | L | L | L | L |
| Uehara T/2012 | L | L | L | U | L | L | L |
| Booth SJ/ 2008 | L | L | L | U | L | L | L |
| Shimada K/2017 | L | L | L | U | L | L | L |
| Zhang H/2019 | L | L | L | H | L | L | L |
| Zhang P/2012/ | L | L | L | U | L | L | L |
| Li W/2011 | L | L | L | U | L | L | L |
| Fan X/2015 | L | L | L | U | L | L | L |
| Sohaib SA/2003 | L | U | L | U | L | L | L |
| Gity M/2019 | L | L | L | U | L | L | L |
| Pereira PN/2018 | L | L | L | U | L | L | L |
| Van TP/2007 | L | L | L | U | L | L | L |
| Thomassin NI/ 2020 | L | L | L | U | L | L | L |

Notes: L=low; U=unclear; H=high.
